# NSG Mice Lacking *Apoe*: An Immunodeficient Mouse Model of Atherosclerosis

**DOI:** 10.1096/fj.202603399R

**Published:** 2026-10-21

**Authors:** Hannah R. Megathlin, Lisa Burzenski, David A. Coleman, Blaze Albert, Michelle L. Farley, Michael A. Brehm, Dale L. Greiner, Leonard D. Shultz

**Affiliations:** ^1^ The Jackson Laboratory Bar Harbor Maine USA; ^2^ Graduate School of Biomedical Sciences and Engineering University of Maine Orono Maine USA; ^3^ Program in Molecular Medicine, Diabetes Center of Excellence UMass Chan Medical School Worcester Massachusetts USA

**Keywords:** Apolipoproteins E, atherosclerosis, humans, hypercholesterolemia, immune system, inbred NOD, mice

## Abstract

Murine research on atherosclerosis has been conducted largely on the C57BL/6 (B6) strain background, and most commonly, the B6.129P2‐*Apoe* ^*tm1Unc*^/J (B6‐ApoE^KO^) strain. The use of the B6 background strain limits translatability for immunotherapy development due to inherent species differences between human and mouse immune systems. A proposed solution to this limitation is a humanized mouse model of atherosclerosis that would support the engraftment of human lymphoid and myeloid cell populations. The development of such a model has been hindered by the lack of a mouse strain that both develops atherosclerosis and supports human immune cell engraftment. In this paper, we report on a NOD.Cg‐*Prkdc* ^*scid*^ *Il2rg* ^*tm1Wjl*^/Sz (NSG) immunodeficient mouse strain that has a 383 bp deletion in the *Apoe* gene which prevents ApoE protein expression. When maintained on a high fat diet, NSG‐ApoE^KO^ mice develop hypercholesteremia and atherosclerosis at levels similar to B6‐ApoE^KO^ mice. While atherosclerosis in B6‐ApoE^KO^ mice has been widely studied in many laboratories under multiple conditions, our initial study of disease development in NSG‐ApoE^KO^ mice was limited and future comparative studies with B6‐ApoE^KO^ mice are necessary. NSG‐ApoE^KO^ mice concomitantly support human cell engraftment while developing atherosclerosis, thereby making them useful to study human‐specific immune aspects.

## Graphical abstract

Immunodeficient NSG mice lacking the Apoe gene develop atherosclerosis when placed on high fat diet (HFD). These mice support engraftment with human CD34+ hematopoietic stem cells. The NSG‐ApoE^KO^ mice can be used in humanized mouse models to examine human‐specific immune disease mechanisms. Created in BioRender.

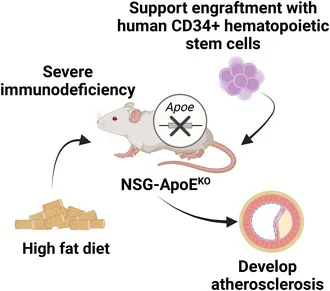

## 1 Introduction

Atherosclerosis is a lipid‐driven chronic inflammatory disease of medium and large blood vessels [1, 2]. Due to the inflammatory nature of the disease, there is growing interest in targeting the immune system in atherosclerosis, with preliminary studies showing promising results [1, 3, 4, 5, 6, 7, 8]. However, no breakthrough treatments in this area have emerged, which may be, in part, due to the lack of predictive preclinical models to develop and test these immunotherapies. The majority of research using animal models of atherosclerosis has utilized immunocompetent mice [9, 10, 11]. While a substantial amount of information about atherosclerosis pathology has been gleaned from these models, they leave a gap for clinical translatability due to inherent species differences in the immune systems between mice and humans [8, 12]. This translatability gap can be bridged by humanized immune system mouse models that use severely immunodeficient mice that support engraftment with human immune cell populations and tissues [13].

Most of the murine research into atherosclerosis has been performed with either the B6.129P2‐*Apoe* ^*tm1Unc*^/J (B6‐ApoE^KO^) or B6.129S7‐*Ldlr* ^*tm1Her*^/J (B6‐LDLR^KO^) strains [10, 11, 14]. Both of these strains were developed through the placement of neomycin resistance cassettes in the targeted genes of 129‐derived embryonic stem cells and implanted into B6 females [15, 16]. The resulting mice were backcrossed for at least 10 generations to the B6 strain. Genetic manipulation was necessary as mice do not spontaneously develop atherosclerosis due to their lipid profiles. Mice lack the cholesterol ester transfer protein (CETP), which facilitates the transformation of atheroprotective high density lipoprotein (HDL) to atherogenic low‐density lipoprotein (LDL) [17]. Apolipoprotein E (ApoE) is associated with very low‐density lipoprotein (VLDL) and binds with high affinity to the low density lipoprotein receptor (LDLR) [18]. When *Apoe* is knocked out, there is an accumulation of lipoprotein remnants which causes hyperlipidemia. Circulating LDL cholesterol is elevated in *Ldlr* knockout mice [9]. The increased circulating cholesterol leads to atherosclerosis development, with complex lesions forming by 20 weeks on high fat diet (HFD) [10, 11]. A more recently developed mouse model utilizes adeno‐associated viral vector (AAV) encoding a gain‐of‐function proprotein convertase subtilisin/kexin Type 9 (PCSK9) [19]. This model allows for the induction of hypercholesterolemia in numerous strains without the need for genetic manipulations or complex breeding schemes to develop new models. This approach causes more moderate increases in cholesterol, similar to mice lacking *Ldlr* [19].

Mouse models have vastly informed our understanding of atherosclerosis pathology and etiology; however, they do not fully recapitulate human disease, which limits their utility for therapeutic development [10, 14]. Humanized mouse models could provide the necessary foundation to develop these therapies [13, 20, 21]. Humanized mouse models employ severely immunodeficient mice that support engraftment with human immune cell populations [13, 22]. NOD.Cg‐*Prkdc* ^*scid*^ *Il2rg* ^*tm1Wjl*^/SzJ (NSG) mice are the most widely used strain for engrafting with human lymphoid or myeloid cells [13, 20]. NSG mice are non‐obese diabetic (NOD) mice that possess two key mutations, *Prkdc* ^*scid*^ and *Il2rg* ^*tm1Wjl*^, that make them highly permissive to human hematopoietic stem cell (HSC) engraftment [23, 24]. The *Prkdc* ^*scid*^ mutation leads to a defect in DNA repair that limits significantly the formation of mature T and B cells [25, 26]. The *Il2rg* ^*tm1Wj*^ mutation inactivates the IL2 common receptor 𝛾‐chain which prevents high affinity signaling through six different cytokine receptors (IL2, IL4, IL7, IL9, IL15, and IL21), functionally blocking IL15‐dependent NK cell development [24]. NSG mice also express a human‐like signal regulatory protein‐⍺ (SIRP‐⍺) that prevents the phagocytosis of human cells, thereby further enhancing human cell engraftment [27].

The development of an atherosclerotic NSG mouse model has been a challenge with the genetic profile of the NOD background. NOD mice have been shown to be resistant to atherosclerosis [28, 29, 30]. It has been reported that neither NOD‐ApoE^KO^ nor NOD‐LDLR^KO^ mice develop atherosclerosis following 12 weeks on high fat diet [30]. Furthermore, NSG mice that have been injected with PCSK9‐AAV develop hypercholesterolemia but fail to develop atherosclerosis [31]. This is in contrast to B6 mice, which develop both hypercholesterolemia and atherosclerosis when treated with a PCSK9‐AAV [19]. In this paper, we describe an immunodeficient mouse model of atherosclerosis in which the *Apoe* gene was knocked out directly in NSG mice. NSG‐ApoE^KO^ mice develop atherosclerosis on HFD and support engraftment with human immune cell populations.

## 2 Methods

### 2.1 Generation of NSG‐ *Apoe* ^*em2Sz*^ Mice (Abbreviated as NSG‐ApoE^KO^ Mice)

All experiments were done under the approval of the Animal Care and Use Committee of The Jackson Laboratory. The mice were housed in elevated barrier pathogen‐free housing at bio‐safety Level 1 status with a 12 h light/12 h dark cycle at 68°F–72°F temperature. The strains used for these experiments were the NOD.Cg‐*Apoe* ^*em2Sz*^ *Prkdc* ^*scid*^ *Il2rg* ^*tm1Wjl*^/SzJ (NSG‐ApoE^KO^), NOD.Cg‐*Prkdc* ^*scid*^ *Il2rg* ^*tm1Wjl*^/Sz (NSG; JAX Strain #: 005557), and B6.129P2‐*Apoe* ^*tm1Unc*^/J (B6‐ApoE^KO^; JAX Strain #: 002052). Exon 3 of the *Apoe* gene was targeted in NSG mice using CRISPR‐Cas9 to create NSG‐ApoE^KO^ mice. Two upstream and two downstream RNA guides were used. The upstream guides were: 5′‐TCAGGACCTACAGTCTTTTT‐3′ and 5′‐GGGTCTCTTTGAAGTGGGTC‐3′. The downstream guides were: 5′‐ATCCAGGGTTGCCTCCTATC‐3′ and 5′‐ACCCAGATAGGAGGCAACCC‐3′. Guides were electroporated into the zygotes as previously published [32]. The targeted *Apoe* allele was fixed to homozygosity. The strain was maintained by sibling matings of homozygous breeder pairs.

### 2.2 PCR and Sequencing

DNA was extracted from livers using the DNeasy genomic DNA preparation kit (Qiagen USA, Germantown, MD). Primers were checked for specificity using BLAST (https://blast.ncbi.nlm.nih.gov/Blast.cgi) [33]. For the knockout allele, the forward primer is 5′‐GGCCTTGATTTCCCCTAACT‐3′ and the reverse primer is 5′‐CAAGAAGGGGCAGGTGT‐3′ which amplifies a 253 bp product. For the wildtype allele, the forward primer is 5′‐AGATCAGCTCGAGTGGC‐3′ and the reverse primer is 5′‐GCTGGACACTCACGTCA‐3′ which amplifies a 151 bp product. Primers were combined with Phusion High Fidelity DNA Polymerase (New England Biolabs Inc., Ipswhich, MA, USA), cresol red, nuclease‐free water, and deoxynucleotides, cycled on a C1000 thermal cycler (Bio‐Rad Laboratories Inc., Hercules, CA, USA) and run out on 2% agarose gels.

### 2.3 Western Blot

Male and female NSG and NSG‐ApoE^KO^ mice were euthanized using carbon dioxide. Liver was removed and dissected into small (~1 mg) pieces. Samples were homogenized in 1 μL per mg of tissue of RIPA lysis buffer (Thermo Fisher Scientific, Waltham, MA, USA; Cat. #89900) containing protease inhibitor tablets (Sigma‐Aldrich Inc., St. Louis, MO, USA; Cat. #11836153001) using pellet pestles (Thermo Scientific; Cat. #12‐141368). Homogenized tissue was incubated on ice for 30 min before centrifugation at 10 000 g for 20 min at 4°C (Eppendorf SE Hamburg, Germany; Cat. #5418 R). Supernatant was isolated and stored at −80°C.

The protein concentration of extracted samples was determined using the Detergent Compatible (DC) Protein Assay (Bio‐Rad; Cat. #500112). Samples were diluted 1:100 with RIPA buffer and prepared using technical duplicates. Absorbance was read on a Spectramax i3 plate reader (Molecular Devices, San Jose, CA, USA). Protein concentrations were determined using a standard curve across a dilution range of 0–1.44 mg/mL of bovine serum albumin (BSA).

ApoE expression was analyzed using the JESS capillary‐based automatic western blot system (ProteinSimple, San Jose, CA, USA; Cat. #004‐650). Protein concentration was normalized so that 12 μg of protein was run for each sample. The assay was executed per the manufacturer's instructions using the 12–230 kDa separation module (ProteinSimple; Cat. #SM‐W003). Primary antibodies raised in rabbit for ApoE (Abcam Inc., Waltham, MA, USA; Cat. #ab183597, 1:200 dilution) and GAPDH (Novus Biologicals, Centennial, CO, USA; Cat. #NB‐10056875, 1:1000 dilution) were used. Proteins were detected using the Anti‐Rabbit Detection Module (ProteinSimple; Cat. #DM‐001).

### 2.4 Treatment With High Fat Diet

Cohorts of 3 male and 3 female mice at 4‐weeks of age were placed on HFD (42% kcal from fat, Inotiv Inc., West Lafayette, IN, USA; Cat. #TD.88137) for 10–28 weeks. Mice were weighed and the diet was changed weekly. Mice maintained on a high fat diet were kept in a high barrier status room that was free from pathogens.

### 2.5 Serum Collection and Lipid Analysis

Blood was collected in serum separator tubes (Greiner Bio‐One GmbH, Kremsmünster, Austria; Cat. #450472), allowed to clot for at least 30 min at room temperature, and spun in an Eppendorf 5418R refrigerated centrifuge at 10 000 g for 10 min. Serum lipid analysis was performed using a Beckman Coulter DxC 700 AU chemistry analyzer (Beckman Coulter Inc., Brea, CA, USA).

### 2.6 Histology

Mice were euthanized using carbon dioxide. Tissues were excised and immediately placed in 10% neutral buffered formalin (NBF) and fixed for 48 h at room temperature. Tissues were trimmed and the aortic root was isolated from hearts [34]. Tissues were embedded in paraffin, sectioned at 5 μm, and stained with hematoxylin and eosin (H&E). Aortic roots were stained with Masson's trichome (MTC).

Automatic immunohistochemistry (IHC) for mouse CD45 (BD Pharmingen, Milford, MA, USA, Cat. #550539, RRID:AB_2177726) in the aortic roots, for F4/80 (Abcam Cat. #ab6640, RRID:AB_1140040; 1:200 dilution) in the lungs and ears, and for human CD45 (Abcam Cat. #ab40763, RRID:AB_726545; 1:250 dilution) in the spleen of engrafted mice was performed using Leica BOND‐RX (Leica Welt, Wetzlar, Germany RRID:SCR_025548). In addition, lungs were stained for Prussian blue. Aortic root sections were stained with rabbit anti‐CD68 (Abcam, Cat. #ab303565, RRID:AB_3075482) and rabbit anti‐⍺ smooth muscle Actin (SMA, Abcam, Cat. #ab5694, RRID:2223021), ImmPRESS horse anti‐rabbit IgG reagent (Vector Laboratories, Plain City, OH, USA, Cat. #MP‐7401, Vector Laboratories Cat. #MP‐7401, RRID:AB_2336529) and ImmPACT 3,3′‐diaminobenzidine (DAB) substrate (Vector Laboratories, Cat. #SK‐4105, Vector Laboratories Cat. #SK‐4105, RRID:AB_2336520). All slides were imaged at 20× using a Hamamatsu NDP Nanozoomer HT2.0/S210 histology slide scanner (Hamamatsu Corporation, Bridgewater, NJ, USA, RRID:SCR_023760).

To isolate aortas, mice were euthanized as above. Organs in the abdominal and thoracic cavities were removed, except for the heart. The aorta was gently detached from the spine while leaving the heart and iliac arteries intact. The torso was then placed in 10% NBF overnight. As much adventitial fat was removed as possible before aortas were excised out from the level of the coronary arteries to the level of the abdominal iliac bifurcation. To stain the aortas, they were transferred to a wax covered petri dish, cut down the middle to expose the interior, and pinned open. A stock solution of oil red O was prepared by dissolving 1 g in 100 mL of 60% isopropyl alcohol. The working solution was made by diluting the stock solution 3:2 with deionized water and filtering through a 0.22 μm filter. Samples were stained with the working solution for 10 min at room temperature. After staining, aortas were washed three times with 60% isopropanol for 3 min [35]. Immediately after staining, aortas were put in PBS and photographed. Images were acquired with a Nikon D7500 (Nikon Inc., Melville, NY, USA) camera with an AF‐S Micro NIKKOR 85 mm lens using the DigiCamControl (https://digicamcontrol.com/) Focus Stacking feature, which takes images at various focus points. Approximately 70 images were acquired per aorta. The images were loaded into Photoshop (Adobe, San Jose, CA, USA) as layers and then aligned and blended so that the area in focus for each layer was visualized.

### 2.7 Plaque Analysis

To determine the area of the aortic root that was obstructed by plaques, images were annotated using the NDP.view2 software (Hamamatsu Corporation, Bridgewater, NJ, USA). The area of the lumen and the plaques was measured. The total area of the lumen was divided by the sum area of the plaques and multiplied by 100 to determine the percent of the lumen that was obstructed by plaques. This was carried out for a minimum of three sections for each mouse, and results were averaged. The person performing this analysis was blinded to the mouse identity. Plaques were isolated from surrounding tissue using Fiji. The percent fibrosis as determined by collagen content was assessed using the FibroSoft software [36].

### 2.8 HSC Engraftment

Groups of 3 female and male NSG‐ApoE^KO^ and NSG controls at 8–12 weeks of age were irradiated with 200 cGy with an X‐RAD320 [37]. Irradiated mice were injected intravenously with 1 × 10^5^ CD34+ HSC from human umbilical cord blood (UCB) or saline as a control [38]. UCB was obtained in accordance with the Committee for the Protection of Human Subjects in Research guidelines of the University of Massachusetts Memorial Umbilical Cord Blood Donation Program as deidentified specimens. Mice were fed normal diet for 4 weeks before mice in the HFD groups were transferred onto the 42% fat diet. All engrafted mice were maintained in a biosafety Level 2 mouse room as described above.

### 2.9 Statistical Analyses

Statistical analysis was performed using GraphPad Prism version 11.0.2 for Mac (GraphPad Software, Boston, MA, USA, RRID:SCR_002798).

## 3 Results

### 3.1 NSG‐ApoE^KO^ Mice Possess a 383 Bp Deletion in Exon 3 of the *Apoe* Gene That Prevents ApoE Expression

Following CRISPR‐Cas9 targeting, 1 NSG female founder was generated with a 383 bp deletion that removed the entirety of exon 3 of the *Apoe* gene (Figure 1A). This mouse was mated with an NSG male, and 2 lines were established from the resulting generation. Both NSG‐ApoE^KO^ L1 and the NSG‐ApoE^KO^ L2 mice lacked the *Apoe* gene when tested via PCR (Figure 1B). Automatic Western blot using the Simple Western demonstrates that neither line produced ApoE protein (Figure 1C). As both lines of the NSG‐ApoE^KO^ mice were determined to be identical in both gene sequencing as well as their lack of expression of ApoE protein, we pooled data from both lines for all future experiments.

**FIGURE 1:**
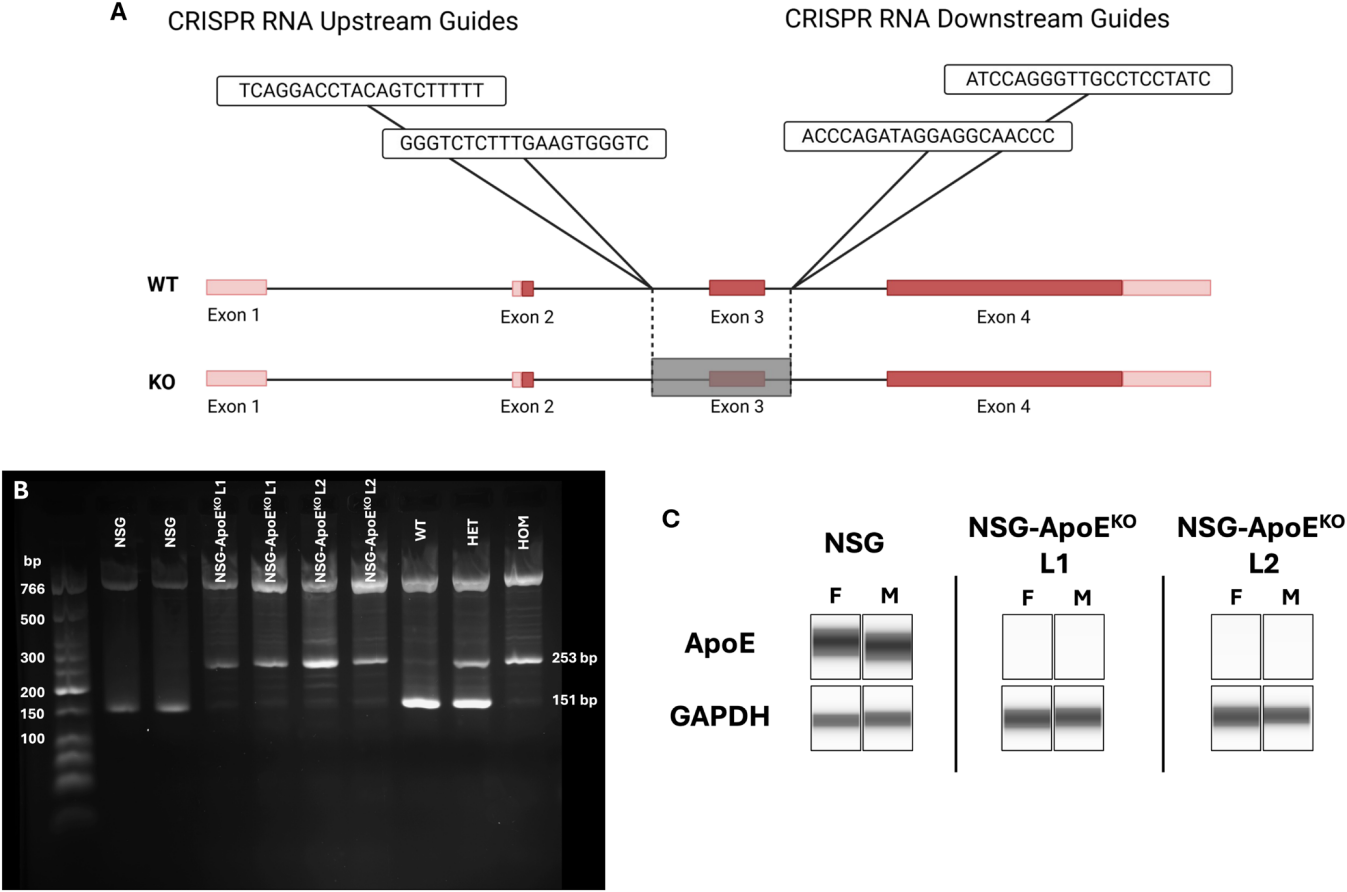
NSG‐ApoE^KO^ mice lack 383 bp in exon 3 of the *Apoe* gene, which leads to no expression of the ApoE protein. Two lines of NSG‐ApoE^KO^ mice were established that lacked 383 bp that removed the entire exon 3 of the *Apoe* gene. Created in BioRender (A). Gel electrophoresis of PCR for *Apoe* knockout bands (253 bp) and wild type (WT) bands (151 bp). Controls for mice homozygous (HOM) and heterozygous (HET) for the *Apoe* knockout allele were used (B). Simple Western identifying lack of ApoE expression in the NSG‐ApoE^KO^ lines. GAPDH sample standards were run at the same time under the same conditions (C).

### 3.2 NSG‐ApoE^KO^ Mice Develop Hypercholesterolemia When Maintained on HFD at Both 8 and 28 Weeks

After confirming that the NSG‐ApoE^KO^ mice do not express ApoE, we compared their serum lipids with NSG and B6‐ApoE^KO^ controls. B6‐ApoE^KO^ mice are the most well‐established mouse model of atherosclerosis and show a sharp increase in serum total cholesterol and LDL in response to HFD treatment [9, 10, 11]. NSG mice with an intact *Apoe* gene were anticipated to have normal cholesterol levels. Due to the reported resistance of both the NOD‐ApoE^KO^ and NOD‐LDLR^KO^ strains to dyslipidemia following HFD treatment, we hypothesized that the NSG‐ApoE^KO^ strain would be similarly resistant, and would therefore only show a mild increase in cholesterol on HFD [30]. However, we found that the NSG‐ApoE^KO^ mice had significantly higher cholesterol (Figure 2A, *p* = 0.0028, Tukey's multiple comparison test) and triglycerides (Figure 2D, *p* = 0.036, Tukey's multiple comparison test) than the B6‐ApoE^KO^ mice following 8 weeks on HFD. When compared to NSG controls, NSG‐ApoE^KO^ mice had a 10‐fold increase in total cholesterol and a 30‐fold increase in LDL (Figure 2A,B). HDL levels were also higher in NSG‐ApoE^KO^ mice than NSG controls, but the increase was ~1.5‐fold (Figure 2C).

**FIGURE 2:**
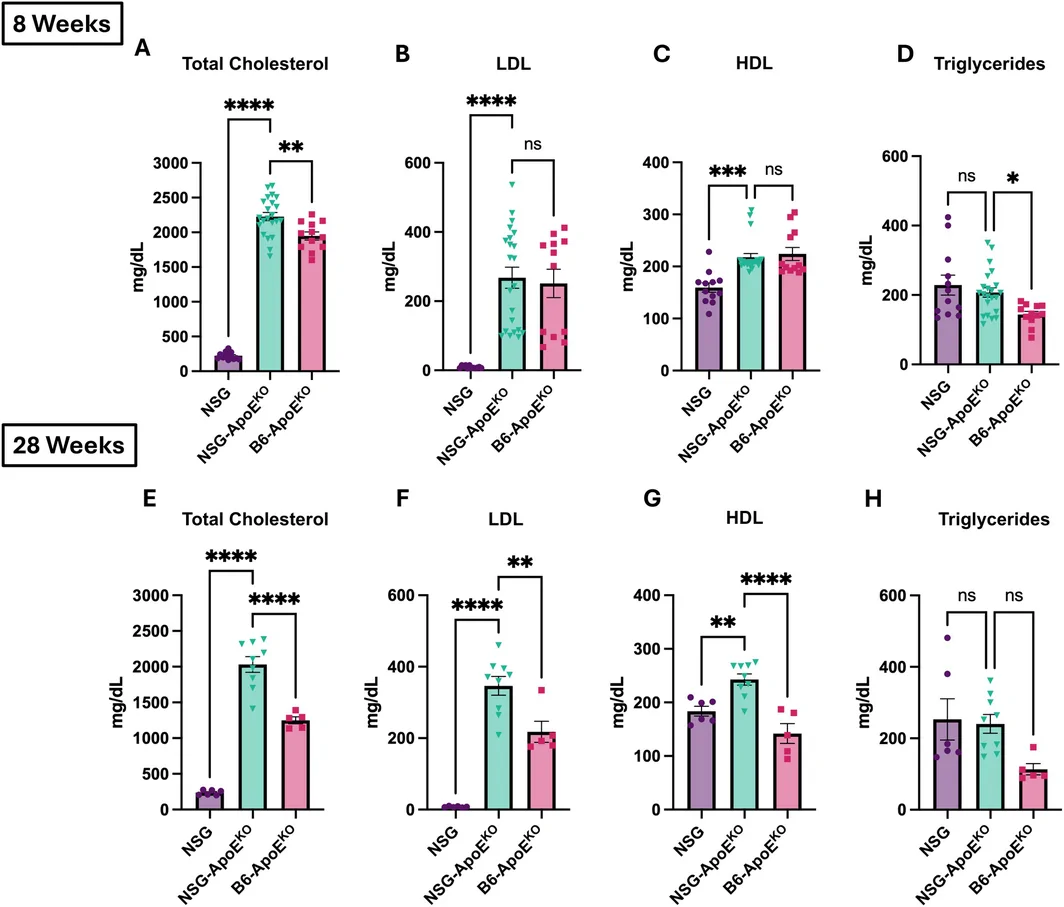
NSG‐ApoE^KO^ mice exhibit hypercholesterolemia when maintained on a high fat diet (HFD) for 8 weeks and 28 weeks. Total cholesterol (A, E), LDL (B, F), HDL (C, G), and triglycerides (D, H) were measured in the sera from 12‐(A‒D, *n* = 12) or 32‐(E‒H *n* = 6) week‐old mice fed high fat diet (42% kcal from fat). Data from two separate experiments. Each symbol represents an individual mouse. Significance determined using 1‐way ANOVA, Tukey's post hoc analysis. Error bars represent SEM. ns—*p* > 0.05; *p ≤ 0.05; **p ≤ 0.01; ***p ≤ 0.001; ****p ≤ 0.0001.

To investigate lipid levels over prolonged exposure to HFD, mice were maintained on HFD for 28 weeks. At this point, serum lipids in NSG and NSG‐ApoE^KO^ mice remained similar to those at the earlier time point, but there was a decrease in the B6‐ApoE^KO^ mice in the total cholesterol and HDL levels (Figure 2E–H). Same as the 8‐week time point, there was a significantly higher level of total cholesterol in NSG‐ApoE^KO^ mice relative to B6‐ApoE^KO^ mice (Figure 2E, *p* = 0.0006, Tukey's multiple comparisons test). In contrast to the 8‐week time point, LDL and HDL were both significantly lower in the B6‐ApoE^KO^ mice than in the NSG‐ApoE^KO^ mice (Figure 2F,G). While B6‐ApoE^KO^ and NSG mice largely maintained their LDL levels from 8 to 28 weeks on HFD (Table S1), NSG‐ApoE^KO^ mice showed a ~1.5‐fold increase in LDL compared to the B6‐ApoE^KO^ (Figure 2F). The significant difference between the HDL levels in the NSG‐ApoE^KO^ and B6‐ApoE^KO^ mice after 28 weeks on HFD (Figure 2G) was due to both a slight increase in the NSG‐ApoE^KO^ mice (Table S1) and a modest decrease in the B6‐ApoE^KO^ mice (Table S1). There were no significant differences between any of the strains for triglyceride levels. However, the triglyceride levels in the B6‐ApoE^KO^ mice trended lower than in the NSG‐ApoE^KO^ mice. At the 8‐week time point, the control mice on normal diet showed similar patterns (Figure S1A–D). However, after 28 weeks, the NSG‐ApoE^KO^ had significantly lower LDL than the B6‐ApoE^KO^ mice and significantly higher triglycerides than both the NSG and B6‐ApoE^KO^ mice (Figure S1F,H). Despite the elevated cholesterol and LDL in the mice lacking ApoE, only the B6‐ApoE^KO^ males gained weight while maintained on the HFD (Figure S2).

### 3.3 Atherosclerotic Plaques Develop in the Aortic Roots of the NSG‐ApoE^KO^ Mice

Next, we examined whether NSG‐ApoE^KO^ mice developed atherosclerosis following HFD treatment (Figure 3A–F). After 28 weeks on HFD, there were atherosclerotic plaques in all of the NSG‐ApoE^KO^ (*n* = 11) and B6‐ApoE^KO^ (*n* = 6) mice tested while no plaques were observed in the NSG (*n* = 6) mice (Figure 3G). When entire aortas were analyzed by oil red O staining, plaques in the NSG‐ApoE^KO^ mice preliminarily appeared similar to those in the B6‐ApoE^KO^ mice, but larger experimental groups are needed to confirm this finding (Figure S3).

**FIGURE 3:**
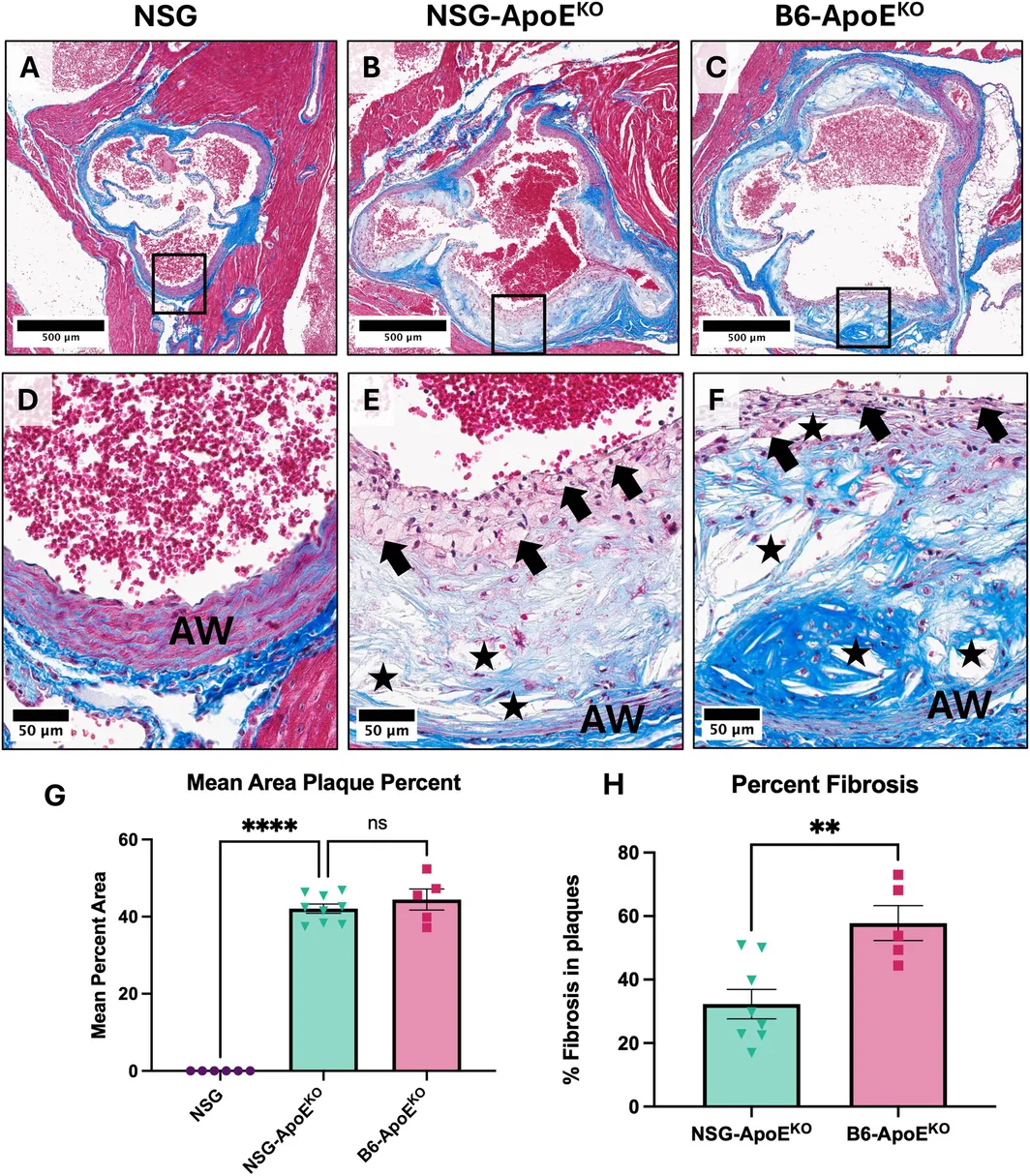
Atherosclerotic plaques are present in the NSG‐ApoE^KO^ mice. (A–D) Representative images of 6 mice of the aortic root from 32‐week‐old NSG (A), NSG‐ApoE^KO^ (B), and B6‐ApoE^KO^ (C) on high fat diet (42% kcal from fat) for 28 weeks. Aortic roots were stained with Masson's trichrome, and slides were imaged with a Hamamatsu NDP Nanozoomer HT.2.0/S210 scanner. Scale bars represent 500 μm. (D–F) Enlarged image of boxes in A–C to visualize walls. AW—aortic wall, arrows indicate foam cells and stars indicate cholesterol clefts. Scale bars represent 50 μm. (G) Mean percent area of the aortic root that is covered by plaques. ****p < 0.0001, 1‐way ANOVA, Tukey's post hoc analysis. (H) Percent fibrosis in the plaques. **p < 0.01, Unpaired *t*‐test.

Morphologically, the plaques in the NSG‐ApoE^KO^ and B6‐ApoE^KO^ mice presented similarly. Both had cholesterol clefts in early necrotic cores, and both were topped by early fibrous caps (Figure 3E,F). Further, the mean area of the lumen that was taken up by plaques was not significantly different between the NSG‐ApoE^KO^ and B6‐ApoE^KO^ mice (Figure 3G). One notable difference between the plaques in the NSG‐ApoE^KO^ and B6‐ApoE^KO^ mice is the amount of collagen in the plaques. In the NSG‐ApoE^KO^ mice, the plaques were identified as 35% collagen, while plaques were comprised of nearly 60% collagen in the B6‐ApoE^KO^ mice (Figure 3H, *p* = 0.0049, unpaired *t*‐test). A plaque's stability comes from its fibrous cap's ability to withstand the force put upon it by blood flow, a key component of which is the collagen content [39, 40, 41]. Staining to visualize immune cells (mouse CD45) macrophages (mouse CD68), and vascular smooth muscle cells (VSMCs, identified by ⍺ smooth muscle actin) in the plaques did not identify any significant differences in the cell content between NSG‐ApoE^KO^ and B6‐ApoE^KO^ mice (Figure 4A–D). Both macrophage and VSMCs were seen in similar patterns in both strains' plaques (Figure 4C,D).

**FIGURE 4:**
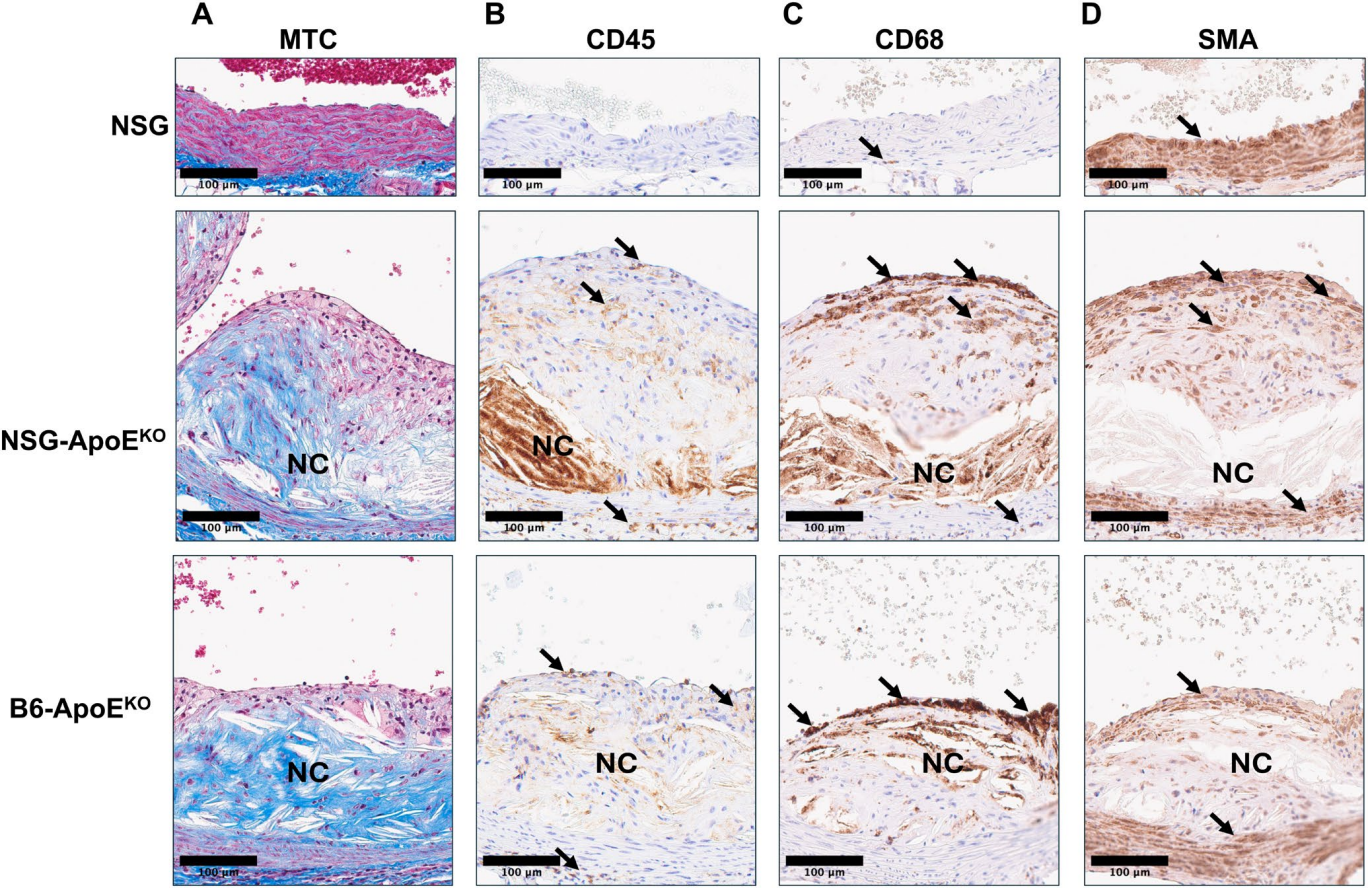
Macrophages and vascular smooth muscle cells are present in atherosclerotic lesions in NSG‐ApoE^KO^ mice. (A) Representative images of 6 mice of the aortic roots stained with Masson's trichrome (MTC). (B–D) Representative images of IHC for CD45 (B), CD68 (C), SMA (D). There is significant background staining in the necrotic cores (NC) for both the mouse CD45 and CD68 sections. Aortic root sections are from 32‐week‐old NSG. NSG‐ApoE^KO^, and B6‐ApoE^KO^ on high fat diet (42% kcal from fat) for 28 weeks. Slides were imaged with a Hamamatsu NDP Nanozoomer HT.2.0/S210 scanner. Scale bars represent 100 μm.

### 3.4 Differences in Lipids Between Male and Female NSG‐ApoE^KO^ and B6‐ApoE^KO^ Mice

It has been established in humans that lipid levels and atherosclerosis risk differ between sexes [42, 43]. In humans, males are at higher risk for cardiovascular disease up until around age 70, at which point the cardiovascular disease incidence switches to being more prevalent in females [44]. Lipid patterns are more complicated and change over time more dramatically than cardiovascular disease risk, but in young and middle‐aged adults, males tend to have higher cholesterol and LDL levels [43]. This has also been seen in the B6‐ApoE^KO^ mice and other mouse models of atherosclerosis [45]. Therefore, we further analyzed our data to determine any sex differences in lipid patterns in the NSG‐ApoE^KO^ mice. After 28 weeks on HFD, there was a significantly higher level of total cholesterol (Figure 5A, *p* < 0.0001, Šídák's multiple comparisons test) and LDL (Figure 5B, *p* = 0.0007, Šídák's multiple comparisons test) in the males than in the females in the NSG‐ApoE^KO^ mice that was not seen in either the B6‐ApoE^KO^ or NSG mice fed the HFD diet. All three strains had significantly lower HDL in the females than in the males (Figure 5C), and no significant differences were seen between the sexes in triglycerides, although there was a trend that they were lower in the NSG and NSG‐ApoE^KO^ females (Figure 5D). After 8 weeks on HFD, the total cholesterol and LDL showed similar patterns, but the HDL levels were not significantly different (Figure S4A–C). The triglycerides were significantly higher in the NSG and NSG‐ApoE^KO^ males at the 8‐week timepoint (Figure S4D). Furthermore, we identified a slight significant difference in the plaque area in the B6‐ApoE^KO^ males compared to the females (Figure 5E). There were no significant differences in the percent of collagen identified between male and females of both NSG‐ApoE^KO^ and B6‐ApoE^KO^ mice (*p* > 0.05, Šídák's multiple comparisons test), although due to limited numbers for comparison, this data is preliminary. The NSG‐ApoE^KO^ trend that the females have less fibrosis than the males (Figure 5F).

**FIGURE 5:**
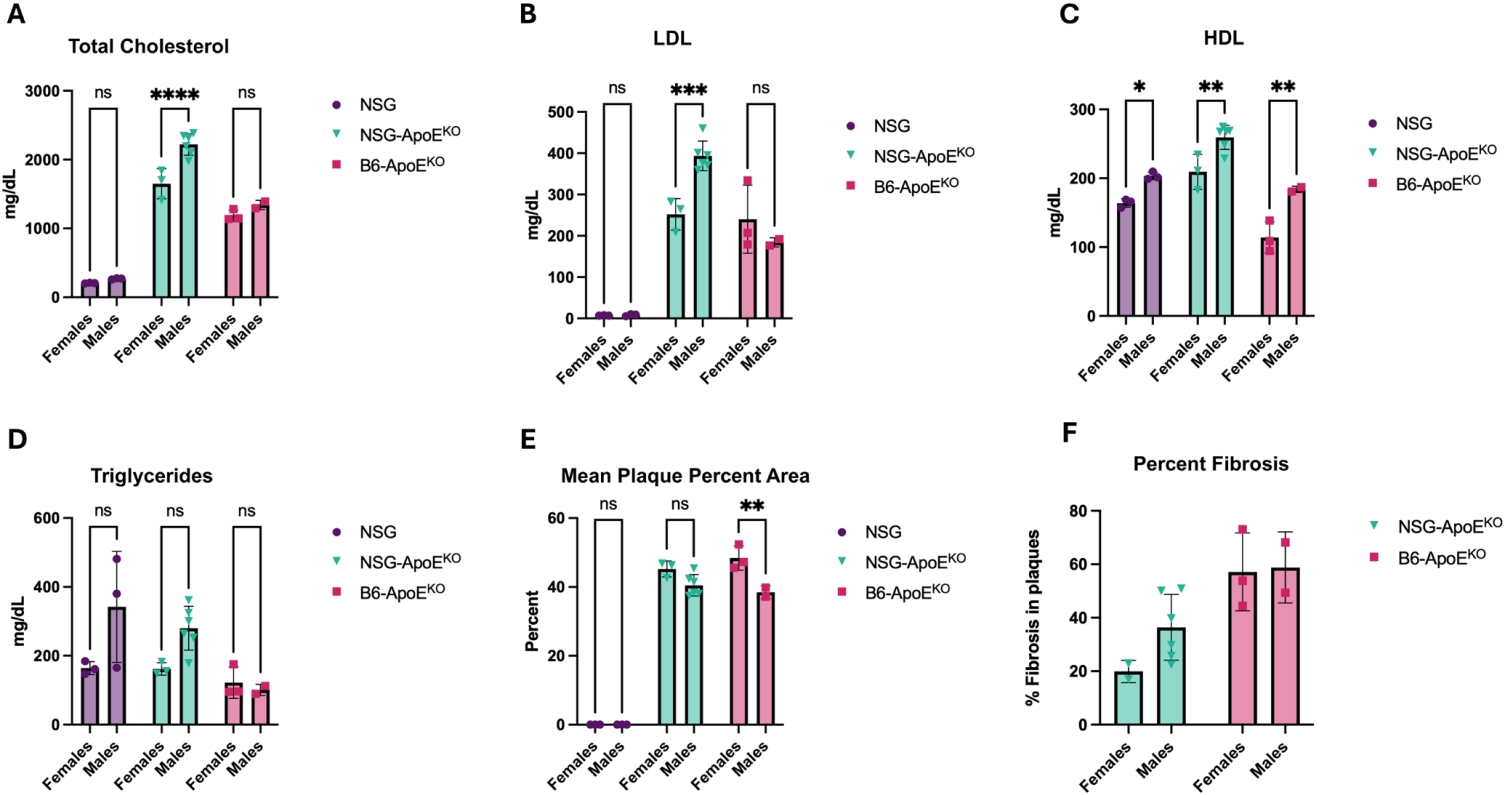
Differences in lipid levels and plaque areas between female and male NSG‐ApoE^KO^ and B6‐ApoE^KO^ mice. Total cholesterol (A), LDL (B), HDL (C), and triglycerides (D) were measured in the sera from 32‐week‐old male and female mice fed high fat diet (42% kcal from fat) for 28 weeks *n* = 6/group. S (E) Mean percent area of the aortic root that is covered by plaques. (F) Percent fibrosis in the plaques. Error bars represent SEM. Significance determined using 2‐way ANOVA, Šídák's post hoc analysis. ns—*p* > 0.05; *p ≤ 0.05; **p ≤ 0.01; ****p ≤ 0.0001.

### 3.5 Lack of ApoE Affects Liver and Skin Morphology in NSG Mice

We further examined effects of the lack of ApoE in other organs of NSG mice maintained on HFD for 28 weeks. Histological analysis uncovered no significant pathologic changes in the kidney or femurs between the NSG, NSG‐ApoE^KO^, and B6‐ApoE^KO^ mice. In the spleen, the only differences noted were in the lack of white pulp and lymphocytes in the NSG and NSG‐ApoE^KO^ mice due to their immunodeficiency (Figure S5).

We examined the lungs of the mice to look for indications of heart failure. Heart failure and atherosclerosis are interconnected diseases with similar comorbidities and risk factors [46]. Macrophages were identified in the lungs of both the NSG and NSG‐ApoE^KO^ mice on HFD. We stained the lungs with Prussian blue, and the absence of positive staining verified that these lung macrophages were not due to heart failure (Figure 6C). When the F4/80 positive cells were quantified in the lungs, there was a significantly higher number of macrophages in the lungs of the NSG and NSG‐ApoE^KO^ mice compared to the B6‐ApoE^KO^ mice (Figure 6D, *p* < 0.001, Tukey's multiple comparisons test).

**FIGURE 6:**
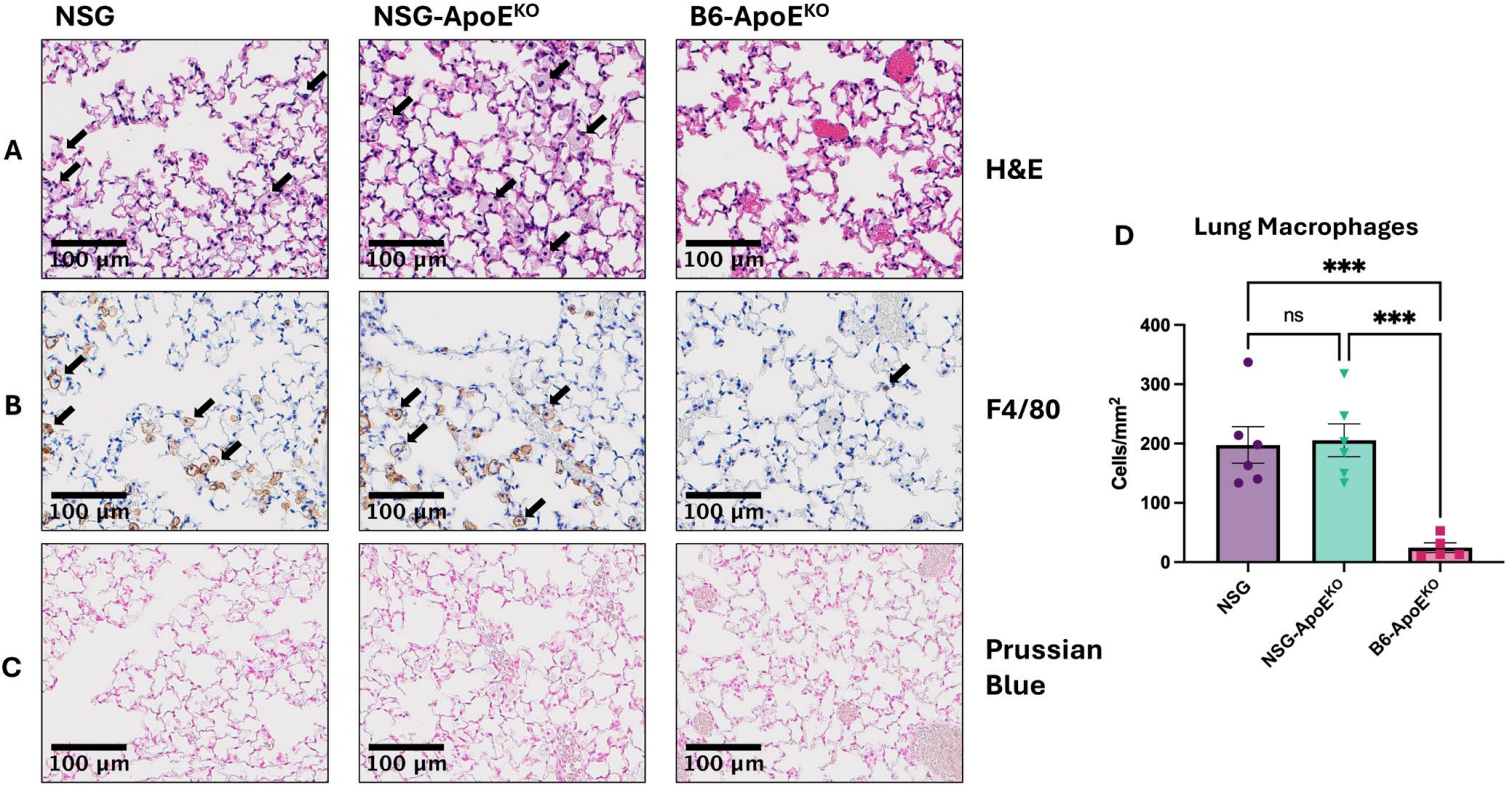
Presence of alveolar macrophages in the lungs of NSG and NSG‐ApoE^KO^ mice. H&E (A), F4/80 IHC (B, brown), and Prussian blue (C) images of lungs from 32‐week‐old NSG (*n* = 6), NSG‐ApoE^KO^ (*n* = 6) and B6‐AopE^KO^ (*n* = 5) mice maintained on high fat diet (42% kcal from fat) for 28 weeks. Arrows indicate macrophages. Scale bars represent 100 μm.(D) Macrophages were quantified from the F4/80 images and the number of macrophages per mm^2^ of lung was calculated. Significance determined using Tukey's post hoc analysis. Error bars represent SEM. ns—*p* > 0.05; ***p ≤ 0.001.

Furthermore, significant differences were noted in the livers between the strains of mice. In the NSG mice on HFD, microvesicular steatosis was observed. In the B6‐ApoE^KO^, macrovesicular steatosis was more prominent. However, in the NSG‐ApoE^KO^ mice, the liver morphology appeared relatively normal, comparable to NSG mice on normal diet. When stained with Masson's trichrome, none of the mice showed significant amounts of fibrosis (Figure 7A,B).

**FIGURE 7:**
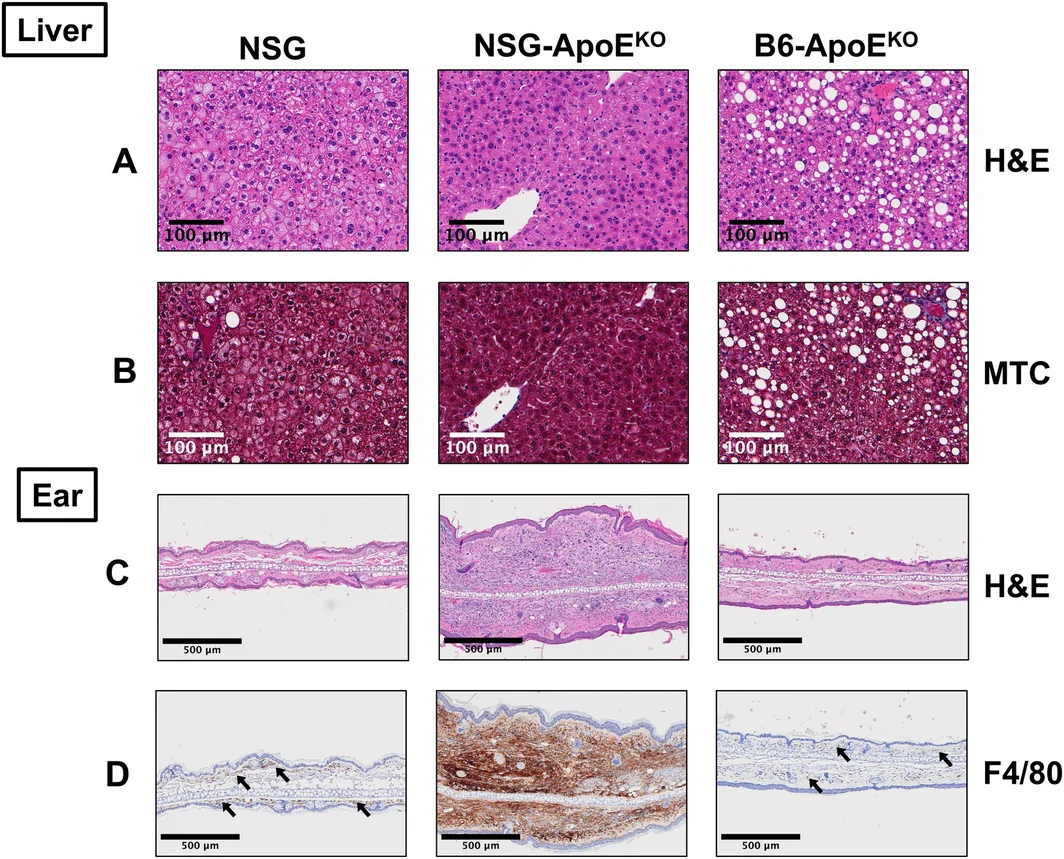
No steatosis in the livers and macrophage infiltration in the ears observed in NSG‐ApoE^KO^ mice on HFD for 28 weeks. Representative images of 6 livers stained with H&E (A) and Masson's trichrome (B; MTC) from 32‐week‐old female NSG, NSG‐ApoE^KO^ and B6‐ApoE^KO^ mice on high fat diet (42% kcal from fat) for 28 weeks. Representative images of ears from 6 mice stained with H&E (C) and IHC for F4/80 (D, brown).

Skin from the ears of mice was also analyzed and showed marked differences. Ears from the NSG‐ApoE^KO^ mice exhibited signs of chronic inflammation as demonstrated by mononuclear cell infiltration and thickening of both the dermal and epidermal layers when compared to NSG and B6‐ApoE^KO^ mice (Figure 7C). IHC for F4/80 identified that the inflammation was composed predominantly of macrophages (Figure 7D).

### 3.6 NSG‐ApoE^KO^ Mice Support Human Cell Engraftment

NSG‐ApoE^KO^ mice support engraftment with human cells while still concomitantly developing atherosclerosis. NSG‐ApoE^KO^ mice that have been engrafted with human CD34+ HSCs demonstrate human CD45+ cells populating their spleens 13 weeks post‐engraftment (Figure 8A). Furthermore NSG‐ApoE^KO^ mice maintain their hypercholesterolemic phenotypes following engraftment. Mice that were engrafted for 18 weeks and on HFD for 14 weeks displayed no significant difference in their lipid profiles (Figure 8B) or in the mean plaque area (Figure 8C,D) when compared to control mice that received saline instead of cells.

**FIGURE 8:**
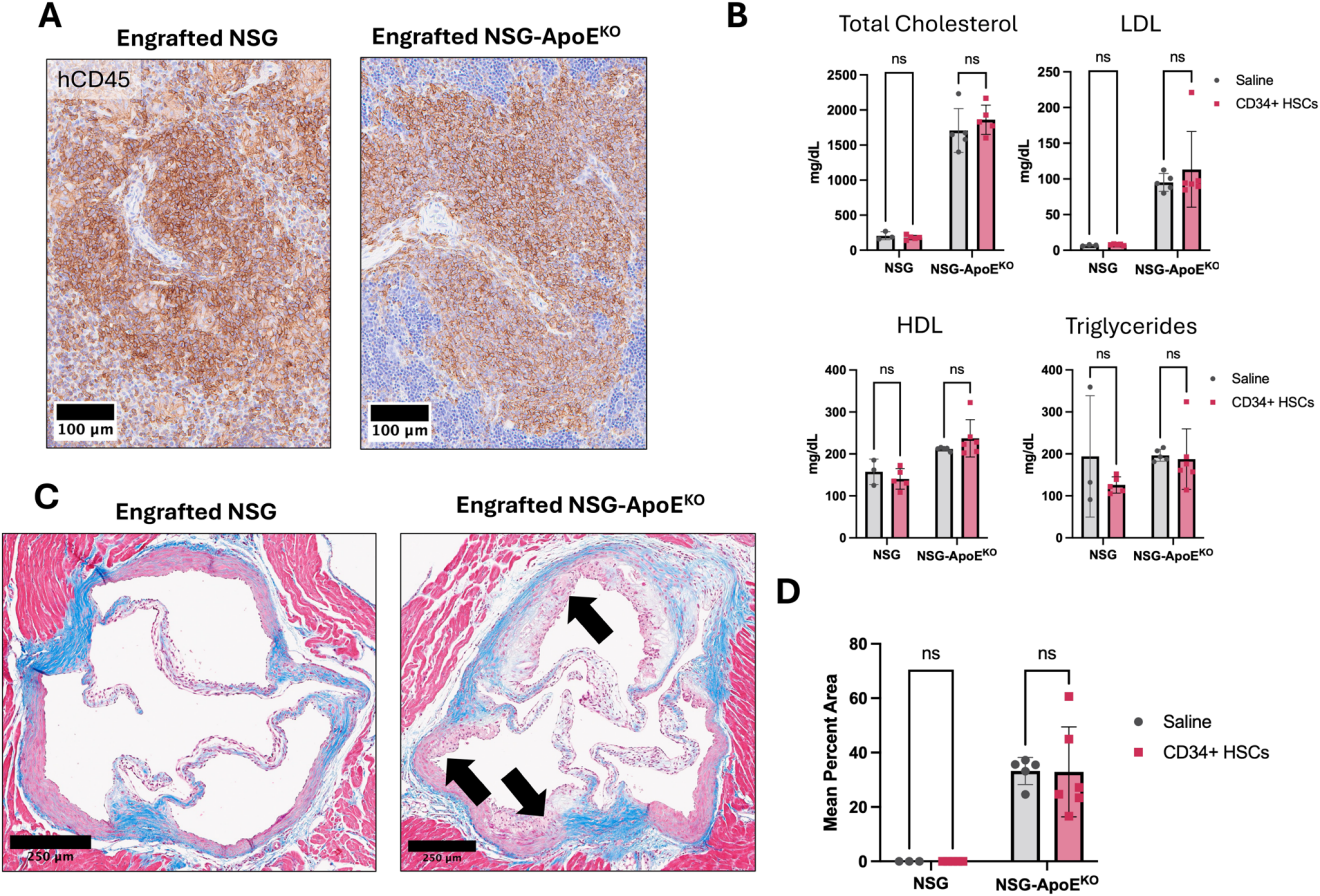
NSG‐ApoE^KO^ support HSC engraftment and develop atherosclerosis in the presence of human immune cells. (A) IHC for human CD45 (hCD45, brown) in the spleen of an NSG and NSG‐ApoE^KO^ mouse engrafted with CD34+ hematopoietic stem cells (HSCs) for 13 weeks. (B) Circulating lipids of NSG and NSG‐ApoE^KO^ mice engrafted with CD34+ HSCs for 18 weeks and on high fat diet (HFD; 42% kcal from fat) for 14 weeks. *n* = 3–6. Error bars represent SEM. Significance determined by Tukey's multiple comparisons test. ns—*p* > 0.05 (C) Representative image of the aortic root from a 23‐week‐old NSG‐ApoE^KO^ 18‐weeks post HSC engraftment on HFD or 14 weeks. Aortic roots were stained with Masson's trichrome, and slides were imaged with a Hamamatsu NDP Nanozoomer HT.2.0/S210 scanner. Arrows indicate plaques. Scale bar represents 250 μm. *n* = 3–6. (D) Mean percent area of the aortic root that is covered by plaques. *n* = 3–6. Error bars represent SEM. Significance determined by Tukey's multiple comparisons test. ns—*p* > 0.05.

## 4 Discussion

Previous studies reported that NOD‐ApoE^KO^ mice fed a HFD do not develop atherosclerosis [28, 30]. NSG‐ApoE^KO^ mice, despite expressing potential NOD atherosclerosis resistance genes and being severely immunodeficient, develop atherosclerosis on HFD. Analysis of the lipid profiles of the NSG‐ApoE^KO^ mice revealed similar profiles to the established B6‐ApoE^KO^ mouse model. Dyslipidemia, in particular the elevation of total cholesterol and LDL, has been strongly correlated with atherosclerosis [2]. In humans, hypercholesterolemia is defined as a fasting serum level greater than 200 mg/dL; however, in mice, these levels are only associated with mild atherosclerosis development in strains that are athero‐prone [47]. Mice can develop hypercholesterolemia when placed on HFD; NOD mice specifically can reach a plasma total cholesterol of 300 mg/dL but still are reported not to develop atherosclerosis [28, 29, 30]. To facilitate the development of atherosclerosis in rodents, knockouts of either the *Apoe* or *Ldlr* genes which specifically raise the level of atherogenic LDL have been used [11, 15, 16]. These models on high‐fat diet exhibit cholesterol levels above 1000 mg/dL and elevated LDL levels, though exactly how much LDL is elevated varies based on intervention [10, 11]. Furthermore, NSG‐ApoE^KO^ mice demonstrate the sex differences in their lipid profiles that are consistent with both the B6‐ApoE^KO^ mice and humans [42, 45, 48, 49]. However, it's worth noting that we used homogenous enzymatic assays to determine the concentration of the LDL and HDL which are clinically approved for human use but are not validated specifically for mouse serum. Although more precise methods such as gas chromatography and mass spectrometry are available, direct enzymatic methods are regularly used in murine atherosclerosis research due to their accessibility and ability for scale [30, 50, 51]. Therefore, our results for LDL and HDL concentrations should be analyzed as representative of overall trends and relationships between experimental groups rather than exact measurements.

Our data indicate that the NSG‐ApoE^KO^ develop atherosclerosis at a level that is comparable to the B6‐ApoE^KO^ mouse, although we cannot establish true equivalence without more extensive lesion quantification than was performed in this preliminary study. This resemblance was unexpected based on the resistance of the NOD strain to develop atherosclerosis reported in the literature [28, 29, 30]. Both NOD‐ApoE^KO^ and NOD‐LDLR^KO^ mice have been shown to have significantly lower levels of cholesterol and LDL as well as a smaller plaque to total aorta ratio than B6‐ApoE^KO^ mice. However, NOD‐ApoE^KO^ LDLR^KO^ double knockout mice develop atherosclerotic phenotypes similar to those of the B6‐ApoE^KO^ mice [30]. NOD mice are the background strain for the NSG mice, so we initially hypothesized that NSG‐ApoE^KO^ mice would be similarly resistant to atherosclerosis. However, our results indicate that NSG‐ApoE^KO^ mice develop atherosclerotic phenotypes similar to the established B6‐ApoE^KO^ model. These data also suggest the possibility that atherosclerosis resistance in NOD mice is mediated by adaptive or innate immune functions that are defective in NSG mice. Moreover, animal health conditions might play a critical role in the development of atherosclerosis. Our NSG mouse colony is maintained at an elevated barrier status, and the absence of certain mouse pathogens could play a role in their susceptibility to atherosclerosis.

Several other differences in organs of the NSG‐ApoE^KO^ mice as compared to the B6‐ApoE^KO^ and NSG mice were observed. In both the NSG and NSG‐ApoE^KO^ mice numerous foamy macrophages in the lungs were present, but none of them stained with Prussian blue, indicating that they were not siderophages due to left sided heart failure [52, 53]. Pulmonary macrophages have roles both in defense against micro‐organisms and promoting tissue homeostasis, and their presence alone is not indicative of a disease state [54]. Several differences have been identified in NOD mice macrophages which may influence their presence in the NSG and NSG‐ApoE^KO^ mice [55, 56, 57]. Future studies are needed to determine whether the elevated numbers of pulmonary macrophages in the NSG and NSG‐ApoE^KO^ mice are a compensatory measure due to their severe immunodeficiency or a feature of the NOD background strain. Steatosis was identified in the livers of both the NSG and B6‐ApoE^KO^ mice but was not seen in the NSG‐ApoE^KO^ mice fed a HFD. However, no fibrosis was identified in the livers of any of the strains. Macrophage infiltration in the ears was observed in HFD‐fed NSG‐ApoE^KO^ mice, which is similar to a phenotype identified in B6‐LDLR^KO^ mice [58].

The main difference between NOD‐ApoE^KO^ and NSG‐ApoE^KO^ mice is the severe immunodeficiency of the NSG‐ApoE^KO^ mice. The NOD mouse immune system has been extensively investigated due to its use as the predominant mouse model of Type 1 Diabetes (T1D) [59, 60]. In these investigations, several key deficits in NOD immune system mice have been established. Inbred NOD mice are deficient in C5 and have no complement lytic activity [61]. Furthermore, NOD macrophages and dendritic cells appear more immature and produce weak functional responses [26, 56, 62]. There is also decreased NK cell activity in NOD mice [26]. The *Prkdc* ^*scid*^ mutation causes a lack of functional T and B cells, while the *Il2rg* ^*tm1Wjl*^ mutation prevents signaling by cytokines IL2, IL4, IL7, IL9, IL15, and IL21, which further prevents development of functional NK cells [24]. Thus, NSG mice lack an adaptive immune system and have an impaired innate immune system. The adaptive immunodeficiency of the NSG‐ApoE^KO^ mouse may be responsible for the discrepancy between the NOD‐ApoE^KO^ resistance and the NSG‐ApoE^KO^ development of atherosclerosis.

Despite widespread agreement that the immune system may have a critical role in the development of atherosclerosis, previous research on immunodeficiency and atherosclerosis in mice has reached contradictory conclusions. Some studies have found little to no role for T and B cells in atherosclerosis [63, 64], while others have found that when functional lymphocytes are absent, plaques are smaller or non‐existent, suggesting an important role for adaptive immune cells in atherosclerosis development [65, 66]. At least one study found larger lesions in immunodeficient mice compared to immunocompetent controls [51].

There are several possible explanations for the variations in results. However, it should be noted that none of the above studies were carried out using NOD background strains. The majority were using B6 mouse strains while the study that found larger lesions used BALB/c mice. A potential explanation for the contradictory results of immunodeficiency's effects on atherosclerosis is strain background. Most murine research in atherosclerosis, including that investigating immunodeficiency, has been performed on mice on the B6 background [10, 67]. There have been many key differences identified in the immune systems of various inbred mouse strains, any of which have the potential to influence atherosclerosis development [68]. B6 mice have been established to be more prone to atherosclerosis compared to other inbred laboratory mouse strains. They will acquire atherosclerosis when placed on HFD without genetic or pharmaceutical interventions to increase cholesterol, albeit the lesions are very mild and take over a year to develop [47, 69]. One study examined how immunodeficiency affected atherosclerosis development in mice deficient for the *Ldlr* gene. The LDLR^KO^ had been backcrossed to B6 mice for either 4 or 10 generations. In these strains, research focused on the effect of *Rag‐1* or *Rag‐2* deletion to confer immunodeficiency. In the strain that had been backcrossed only four times, it was found that immunodeficiency led to a 65%–70% decrease in atherosclerosis in both the aortic root and innominate artery. However, in the strain that had been backcrossed for 10 generations, the immunodeficiency only led to a reduction in atherosclerosis in the innominate artery. This study highlights how even minor strain differences can alter effects of immunodeficiency on atherosclerosis development [70]. While several reports have indicated either no changes or a reduction in size of plaques with immunodeficiency, those studies have been in B6 strains [63, 64, 65, 70, 71]. In an investigation that utilized BALB/c background mice rather than B6 mice, the plaques were reported to be larger in the immunodeficient mice than in their immunocompetent counterparts following HFD treatment [51]. There are a number of identified differences in the immune systems of B6 and BALB/c mice. B6 mice are known to have a Th1 cell bias compared to BALB/c mice, which skew toward Th2 cells [68, 72]. These differences have been identified to alter immunometabolic phenotypes in mice when they are placed on a HFD, which may be contributing to the contradictory results about the role of the adaptive immune system in atherosclerosis [73].

Atherosclerosis is a disease of aging and therefore animal age and study duration need to be considered when comparing results [14, 74]. Typically, atherosclerosis is studied in mice that are between 3 and 6 months of age, which correlates to young adulthood in humans [75]. Similarly, the duration of the exposure to the elevated cholesterol levels factors into disease burden and complexity [76]. Most studies typically maintain mice on a HFD between 2 and 6 months. Aging in mouse models of atherosclerosis has been shown to exhibit different immune phenotypes than in younger mice under the same conditions [76, 77]. These differing aging phenotypes have been observed in ApoE^KO^, LDLR^KO^, and PCSK9‐AAV models [58, 76, 77, 78]. Bone marrow‐derived macrophages from old mice accumulated more lipid droplets and failed to properly react to lipid loading [79]. Although similar study designs have been used, with similar ages and diet durations, mouse strain also differentially affects aging, which may influence strain susceptibility to atherosclerosis [80, 81]. The C57BL/6 strain is one of the longest‐lived inbred mouse strains and their HSCs maintain their self‐renewal capabilities longer than DBA/2 and BALB/c mice [82].

Environmental conditions are also necessary to consider when evaluating differing results in atherosclerosis studies. A large amount of variability has been identified between studies using the same strains and similar experimental designs [83]. A major contributor to this variability could be the impact of housing conditions on atherosclerosis. It is well established that housing conditions greatly affect mouse immune systems [84, 85]. Our colonies at The Jackson Laboratory are maintained at an elevated barrier status and the experiments described herein occurred in high barrier levels. Both barrier status rooms are free from most pathogens, most notably, *Heliobacter* spp., *Rodentibacter pneumotropicus*, and *Pneumocystis murina*. These pathogens were found to be associated with increased levels of atherosclerosis formation [86]. Additionally, microbiomes beyond pathogenic organisms and other environmental influences can substantially affect atherosclerosis development and are not easily compared between studies [87]. These differences in disease progression could be further exacerbated by immunodeficiency, as immune systems in immunocompetent mice could be activated by the environmental pathogens, while immunodeficient mice lack adaptive responses.

While the lack of clear consensus on whether the adaptive immune system is atherogenic, atheroprotective, or irrelevant in murine atherosclerosis formation could be due to a few different variables, our data support the hypothesis that the adaptive immune system may be atheroprotective. Ongoing experiments are in progress where we have backcrossed the 383 bp deletion in the *Apoe* gene onto the NOD strain to directly compare NOD‐ApoE^KO^ mice with our NSG‐ApoE^KO^ mice under the same experimental conditions, including room and diet. Furthermore, this study will investigate the relationship between T1D and atherosclerosis. NOD mice develop autoimmune‐mediated diabetes starting at 12–14 weeks of age and are the predominant mouse model of T1D [59, 60, 88]. Cardiovascular disease and atherosclerosis have been correlated with T1D, and there are numerous confounders and overlapping pathologies that can be investigated in future studies with our model [89, 90, 91].

The chronic inflammation and hyperlipidemia characteristic of atherosclerosis are also associated with comorbidities outside of the vascular system. One of these comorbidities is metabolic‐associated liver disease (MASLD). There is significant overlap in these disease pathologies; both diseases involve inflammation, hyperlipidemia, and endothelial dysfunction [92]. In the B6‐ApoE^KO^ mice, we identified macrovesicular steatosis. In contrast, the NSG mice on HFD demonstrated microvesicular steatosis, which is associated with more severe disease [93]. However, there was no significant steatosis observed in the NSG‐ApoE^KO^ mice. In studies using B6 background mice, ApoE deficiency has been identified as protective from MASLD [94, 95]. Despite exhibiting microvesicular steatosis, the NSG mice on HFD did not show substantial fibrosis in the liver, which is a limitation for current animal models of MASLD [96]. NSG mice on HFD alone do not provide an improved model for MASLD, but their potential for humanization makes them an area to investigate in future studies.

While NSG‐ApoE^KO^ mice can be used to gain further insight into how the mouse immune system functions in atherosclerosis, their role in future studies of atherosclerosis is supported by their severe immunodeficiency in tandem with a human‐like SIRP‐⍺. NSG mice support high levels of engraftment with hematopoietic human cells or tissues [13, 24]. Our preliminary study indicates that NSG‐ApoE^KO^ mice support human immune cell engraftment while concomitantly developing atherosclerosis, supporting their use in future studies to investigate human immunity and atherosclerosis. This study was limited in the number of mice used as well as the phenotyping performed, so future studies will replicate these results with more robust characterization. Humanized mouse models support a wide variety of research uses including cancer immunotherapy [21]. NSG‐ApoE^KO^ mice may likewise serve as a model for immunotherapy of atherosclerosis. Additionally, these mice can be crossed with next generation NSG models such as the NSG‐SGM3 (JAX Strain #: 013602) or the NSG‐MHC^DKO^ (JAX Strain #: 025216) mice to overcome some of the limitations currently associated with humanized mouse models such as poor myeloid cell engraftment and xenogeneic interactions [97, 98].

## Author Contributions

The research was conceived and designed by Leonard D. Shultz, Hannah R. Megathlin, and Lisa Burzenski. Studies were conducted and data were collected by Hannah R. Megathlin, Lisa Burzenski, Michelle L. Farley, and Blaze Albert. Data analysis and interpretation were performed by Hannah R. Megathlin, Leonard D. Shultz, Lisa Burzenski, and David A. Coleman. Hannah R. Megathlin, Leonard D. Shultz, Lisa Burzenski, David A. Coleman, Michael A. Brehm, and Dale L. Greiner participated in drafting and revising the manuscript.

## Funding

These studies were supported by NIH grants, 1 UG3 DK142192 and 3T32GM132006, Breakthrough T1D grant 4‐COE‐2025‐1751‐A‐N, by the Harvey A. Shultz Humanized Mouse Initiative, and The Jackson Laboratory Director's Innovation Fund.

## Conflicts of Interest

The authors declare no conflicts of interest.

## Supporting information


**Figure S1:** Male and female NSG‐ApoE^KO^ mice exhibit hypercholesterolemia when maintained on normal diet for 8 weeks and 28 weeks.
**Figure S2:** Body weights of mice on normal (NC) and high fat diet (HFD).
**Figure S3:** Whole aorta staining with oil red O from NSG‐ApoE^KO^ and B6‐ApoE^KO^ 32‐week‐old females on HFD for 28 weeks.
**Figure S4:** Differences between sexes in the lipids of mice after 8 weeks on high fat diet (42% fat).
**Figure S5:** No unexpected histological differences between NSG, NSG‐ApoE^KO^ and B6‐ApoE^KO^ kidneys, spleen, and bone marrow.
**Table S1:** Lipid values of experimental mice.

## Data Availability

All data generated or analyzed during this study are included in this published article or in its Supporting Information. Further inquiries can be directed to the corresponding author upon reasonable request.
