# Supplementary material for: NSG Mice Lacking Apoe: An Immunodeficient Mouse Model of Atherosclerosis
Source: FASEB J. 2026 Oct 21;40(20):e72395. doi: 10.1096/fj.202603399R (PMC13686551; doi:10.1096/fj.202603399R)
Supplement: Supplementary file 1 [file FSB2-40-e72395-s001.docx]

**Supplemental Figure 1. Male and female NSG-ApoE^KO^ mice exhibit hypercholesterolemia when maintained on normal diet for 8 weeks and 28 weeks.** Total cholesterol (A, E), LDL (B, F), HDL (C, G), and triglycerides (D, H) were measured in the sera from 12-(A-D, n=12) or 32-(E-H, n=6) week-old mice fed normal chow diet (6% kcal from fat). Data from two separate experiments. Each symbol represents an individual mouse. Significance determined using 1-way ANOVA, Tukey’s post hoc analysis. Error bars represent SEM. ns – P > 0.05; *P ≤ 0.05; **P ≤ 0.01; ***P ≤0.001; ****P ≤ 0.0001

**Supplemental Figure 2. Body weights of mice on normal (NC) and high fat diet (HFD).** Each symbol represents the group mean; n=12. NC - normal chow (6% kcal from fat); HFD - high fat diet (42% kcal from fat)

**Supplemental Figure 3. Whole aorta staining with oil red O from NSG-ApoE^KO^ and B6-ApoE^KO^ 32-week-old females on HFD for 28 weeks.**

**Supplemental Figure 4. Differences between sexes in the lipids of mice after 8 weeks on high fat diet (42% fat).** Each symbol represents an individual mouse, n=6/group

**Supplemental Figure 5. No unexpected histological differences between NSG, NSG-ApoE^KO^ and B6-ApoE^KO^ kidneys, spleen, and bone marrow.** Representative images of kidneys (A), spleens (B), and bone marrow (C) stained with H&E (rom 32-week-old female NSG, NSG-ApoE^KO^ and B6-ApoE^KO^ mice on high fat diet for 28 weeks.

**Supplemental Table 1: Lipid Values of experimental mice**

|  | | | **NC** | | | | **HFD** | | | |
| --- | --- | --- | --- | --- | --- | --- | --- | --- | --- | --- |
| **Strain** | **Sex** | **Weeks on Diet** | **Total Cholesterol ± SEM (mg/dL)** | **LDL ± SEM (mg/dL)** | **HDL ± SEM (mg/dL)** | **Triglycerides ± SEM (mg/dL)** | **Total Cholesterol ± SEM (mg/dL)** | **LDL ± SEM (mg/dL)** | **HDL ± SEM (mg/dL)** | **Triglycerides ± SEM (mg/dL)** |
| NSG | F | 8 | 92.86 ± 1.78 | 5.11 ± 0.58 | 67.73 ± 1.82 | 191.67 ± 6.78 | 179.67 ± 5.67 | 7.89 ± 0.71 | 136.08 ± 6.40 | 158.17 ± 11.79 |
|  |  | 28 | 77.00 ± 5.00 | 4.68 ± 0.39 | 61.43 ± 3.69 | 165.00 ± 17.21 | 206.67 ± 3.18 | 7.08 ± 0.27 | 163.97 ± 3.57 | 164.00 ± 10.79 |
|  | M | 8 | 121.67 ± 2.73 | 2.10 ± 0.42 | 97.65 ± 1.90 | 254.50 ± 28.63 | 266.00 ± 15.14 | 10.47 ± 1.89 | 182.88 ± 9.61 | 299.00 ± 39.17 |
|  |  | 28 | 108.00 ± 4.04 | 1.46 ± 0.17 | 94.30 ± 2.49 | 158.00 ± 29.37 | 268.67 ± 5.24 | 8.44 ± 1.37 | 203.30 ± 3.16 | 342.00 ± 93.18 |
|  | Com | 8 | 105.08 ± 5.24 | 11.06 ± 5.20 | 119.82 ± 9.68 | 220.67 ± 29.21 | 222.83 ± 15.13 | 9.18 ± 1.04 | 159.48 ± 8.95 | 228.58 ± 28.83 |
|  |  | 28 | 92.50 ± 7.50 | 3.07 ± 0.74 | 77.87 ± 7.61 | 161.50 ± 15.30 | 237.67 ± 14.13 | 7.76 ± 0.69 | 183.63 ± 9.05 | 253.00 ± 57.83 |
| NSG-ApoE^KO^ | F | 8 | 697.70 ± 31.42 | 15.43 ± 3.20 | 122.96 ± 4.55 | 197.80 ± 12.55 | 1995.10 ± 59.73 | 248.93 ± 44.85 | 233.42 ± 13.83 | 160.90 ± 11.21 |
|  |  | 28 | 648.25 ± 25.46 | 21.84 ± 4.11 | 102.50 ± 3.33 | 246.50 ± 53.71 | 1649.67 ± 126.19 | 251.85 ± 22.23 | 209.30 ± 14.80 | 161.67 ± 10.37 |
|  | M | 8 | 883.89 ± 40.77 | 15.09 ± 3.19 | 137.29 ± 2.71 | 351.89 ± 31.66 | 2420.92 ± 46.99 | 283.29 ± 42.90 | 204.73 ± 1.75 | 246.08 ± 16.10 |
|  |  | 28 | 946.33 ± 25.72 | 13.48 ± 0.95 | 156.83 ± 7.26 | 385.33 ± 16.33 | 2220.33 ± 64.03 | 393.71 ± 14.64 | 259.40 ± 7.12 | 279.83 ± 26.04 |
|  | Com | 8 | 785.89 ± 33.01 | 15.27 ± 2.20 | 129.75 ± 3.14 | 270.79 ± 24.11 | 2227.36 ± 58.88 | 267.67 ± 30.52 | 217.77 ± 6.92 | 207.36 ± 13.57 |
|  |  | 28 | 776.00 ± 62.50 | 18.26 ± 2.80 | 125.79 ± 11.45 | 306.00 ± 40.61 | 2030.11 ± 109.92 | 346.42 ± 26.26 | 242.70 ± 10.45 | 240.44 ± 26.06 |
| B6-ApoE^KO^ | F | 8 | 807.83 ± 58.07 | 17.09 ± 6.61 | 123.00 ± 10.51 | 141.50 ± 11.73 | 1850.67 ± 62.75 | 276.03 ± 56.78 | 218.97 ± 13.42 | 130.33 ± 15.17 |
|  |  | 28 | 798.00 ± 44.23 | 44.68 ± 5.21 | 85.73 ± 3.84 | 140.67 ± 27.72 | 1187.67 ± 47.27 | 240.36 ± 47.66 | 113.97 ± 13.05 | 122.00 ± 26.50 |
|  | M | 8 | 792.17 ± 52.59 | 6.66 ± 1.69 | 118.35 ± 4.80 | 189.67 ± 14.79 | 2045.17 ± 87.04 | 226.47 ± 62.91 | 229.03 ± 22.26 | 157.00 ± 7.09 |
|  |  | 28 | 978.00 ± 55.87 | 32.02 ± 14.55 | 115.47 ± 13.40 | 188.33 ± 45.90 | 1342.00 ± 48.00 | 184.17 ± 7.87 | 183.85 ± 3.25 | 100.50 ± 11.50 |
|  | Com | 8 | 800.00 ± 37.42 | 11.87 ± 3.61 | 120.68 ± 5.55 | 165.58 ± 11.56 | 1947.92 ± 58.96 | 251.25 ± 41.09 | 224.00 ± 12.48 | 143.67 ± 8.94 |
|  |  | 28 | 888.00 ± 51.34 | 38.35 ± 7.47 | 100.60 ± 14.27 | 164.50 ± 26.24 | 1249.40 ± 48.27 | 217.89 ± 29.61 | 141.92 ± 18.58 | 113.40 ± 15.86 |

NC-Normal chow; HFD – High fat diet; Com – combined.
